# Can large language models help predict results from a complex behavioural science study?

**DOI:** 10.1098/rsos.240682

**Published:** 2024-09-25

**Authors:** Steffen Lippert, Anna Dreber, Magnus Johannesson, Warren Tierney, Wilson Cyrus-Lai, Eric Luis Uhlmann, Emotion Expression Collaboration, Thomas Pfeiffer

**Affiliations:** ^1^ Department of Economics, University of Auckland, Auckland, New Zealand; ^2^ Department of Economics, Stockholm School of Economics, Stockholm, Sweden; ^3^ Department of Economics, University of Innsbruck, Innsbruck, Austria; ^4^ Organisational Behaviour Area/Marketing Area, INSEAD, Singapore; ^5^ Graduate School of Business, Stanford University, CA, USA; ^6^ New Zealand Institute for Advanced Study, Massey University, Auckland, New Zealand

**Keywords:** forecasting, large language models, meta-research

## Abstract

We tested whether large language models (LLMs) can help predict results from a complex behavioural science experiment. In study 1, we investigated the performance of the widely used LLMs GPT-3.5 and GPT-4 in forecasting the empirical findings of a large-scale experimental study of emotions, gender, and social perceptions. We found that GPT-4, but not GPT-3.5, matched the performance of a cohort of 119 human experts, with correlations of 0.89 (GPT-4), 0.07 (GPT-3.5) and 0.87 (human experts) between aggregated forecasts and realized effect sizes. In study 2, providing participants from a university subject pool the opportunity to query a GPT-4 powered chatbot significantly increased the accuracy of their forecasts. Results indicate promise for artificial intelligence (AI) to help anticipate—at scale and minimal cost—which claims about human behaviour will find empirical support and which ones will not. Our discussion focuses on avenues for human–AI collaboration in science.

## 1. Introduction

Anticipating in advance what will happen in an empirical research investigation is an important aspect of scientific research. For the design of empirical research, effect size forecasts are valuable when determining sample size requirements. The expected presence or absence of potential effects from confounding factors and moderators informs sampling strategies and defines which variables to record and include in a statistical analysis. Accurate predictions of empirical results can therefore help allocate resources efficiently to original studies as well as replications [1–4]. In a broader context, accurate forecasts for empirical effects imply a deep understanding of real-world phenomena, providing foundations for applying scientific knowledge to real-world problems.

Accurate predictions for effects in empirical research are often difficult to obtain, in particular when untested hypotheses are investigated, and novel methodologies are employed. The perhaps best guidance comes from prior empirical results and theory, with domain expertise typically being required to correctly anticipate how prior results carry over into a novel experimental setting. Additionally, in the social and behavioural sciences, lay persons might be able to generate accurate predictions based on their own intuition and experiences [5]. Expectations regarding effect sizes and their statistical significance have been elicited from experts in several studies, most notably for large-scale replication projects [1,6–8], but also for studies investigating the generalizability of research claims and the applicability of competing theories to novel empirical settings [2,9–14]. Successful predictions of empirical research outcomes by human forecasters have also inspired the development of computational approaches in this domain [15–17].

Emerging applications of large language models (LLMs) for forecasting tasks [18–20] and for emulating human behaviour [21,22] motivated us to investigate whether LLMs [23] can help generate accurate forecasts for the outcomes of a complex experiment from the behavioural sciences. Similar to other artificial intelligence (AI) tools, LLMs can be used for this task as standalone tools [21,22], and in a human/AI collaborative setting [24–28]. Standalone applications offer the advantage of scalability, as chatbots can be easily and cost-effectively queried about a large number of potential experimental effects. On the other hand, the forecasting of experimental effects may be a well-suited task for a human/AI collaborative setting that allows bringing together complementary strengths [24–28], such as the wealth of encyclopaedic information accessible through LLMs and humans’ personal experiences with social systems and human behaviour.

We used OpenAI’s GPT models [23,29] as leading general purpose LLMs and investigated them as stand-alone forecasting tools (study 1) as well as in a human/AI collaborative setting (study 2). For the remainder of §1, we will first give a description of the forecasting task, and then provide a brief description of study 1 and study 2. A more comprehensive description of the design of both studies is given in §4.

For the forecasting task, we used 24 effect sizes from a recent crowdsourced study (Tierney W, unpublished data) investigating reactions to female and male professionals who express anger in a professional environment. This ‘many-labs’ [30] and ‘many-designs’ [11,31,32] initiative, in the following referred to as the ‘anger expression study’, was conducted in March–June 2021. When we conducted the forecasting study presented here, the 24 effect sizes were known to us, but they were not yet published in any format.

Each participant in the anger expression study evaluated a target individual who responded to a situation in her or his work environment (Tierney W, unpublished data). Using a many-design approach, independent teams of researchers created 27 experimental designs manipulating both emotional expression (anger or no-anger) and the gender of the target (female or male). These included both a direct replication (same materials and methods) of the earlier research finding that anger expressions signal status and competence for men but elicit backlash against women [33], and 26 conceptual replications (same idea, different method). In all of the designs, two key experimental conditions were used: in the ‘anger condition’, the target person is depicted to respond to the incident with anger; in the ‘no-anger condition’ the target responds to the same incident neutrally or with sadness. The 27 designs used a variety of workplace situations as well as different formats (e.g. videos, audio recordings, graphic novel, written vignettes and ostensive newspaper stories) to depict the incident, manipulate the target’s gender, and portray her or his response. A large number of participants (*n* = 7 665, both female and male) from 23 countries were recruited into the experiment. Each participant was randomized into a single between-subjects condition and then evaluated the target person on numeric scales along six characteristics (competence, warmth, being ‘out-of-control’, dominance, assertiveness and accorded status). The difference in these ratings between ‘anger condition’ and ‘no-anger condition’ for the four combinations of *target gender* × *participant gender* defined the 24 effect sizes that are the focus of this forecasting study.

The anger expression study contains a forecasting component, where effect size forecasts were elicited from a cohort of researchers (*n* = 119) through an incentivized survey (see Supplement 17 in (Tierney W, unpublished data); electronic supplementary material; and §4 for further detail). This cohort was able to anticipate the replication results, in that forecasted and observed effect sizes were statistically significantly positively correlated (*r* = 0.87). Yet, the researcher cohort also made systematic errors, in particular overestimating the degree of gender bias that would emerge in the large-scale data collection. They expected backlash effects against angry women [33] as well as stereotype-driven perceptions of women as less competent, assertive and dominant than men, and as less deserving of status. However, such gender biases did not reliably emerge across the many designs and subject populations in the crowdsourced replication initiative.

We present two forecasting investigations examining whether LLMs (GPT-3.5 and GPT-4) can generate accurate effect size predictions for the anger expression study (study 1), and whether human–AI collaboration can improve human forecasts (study 2). In brief, for study 1, we generated ensembles of 50 effect size forecasts with GPT-3.5 (model gpt-3.5-turbo-0613) and GPT-4 (model gpt-4-0613) under default parameter settings as stand-alone tools. Moreover, in a robustness analysis, we also explored the impact of the temperature parameter on GPT-4 model responses (see the electronic supplementary material). For study 2, we implemented a human/AI collaborative setting with a cohort of participants from a university subject pool. Participants in study 2 attended in-person and were randomized into one of the following two experimental conditions. In the ‘ChatGPT setting’, participants provided an initial unaided set of forecasts for the 24 effects. They were then provided access to a GPT-4 powered chatbot and allowed to revise their estimates. In the ‘Internet setting’, participants also provided an initial unaided set of forecasts for the 24 effects. They were then allowed to revise their estimates with the help of information through Internet searches but were not allowed to use AI chatbots. This approach allows us to test if the participants’ revised responses are more accurate than their initial responses, and if this depends on the experimental condition. Study 2 was preregistered with a detailed pre-analysis plan, whereas study 1 was not preregistered.

## 2. Results

### 2.1. Forecasts generated by GPT-3.5 and GPT-4 (study 1)

**Figure 1.**
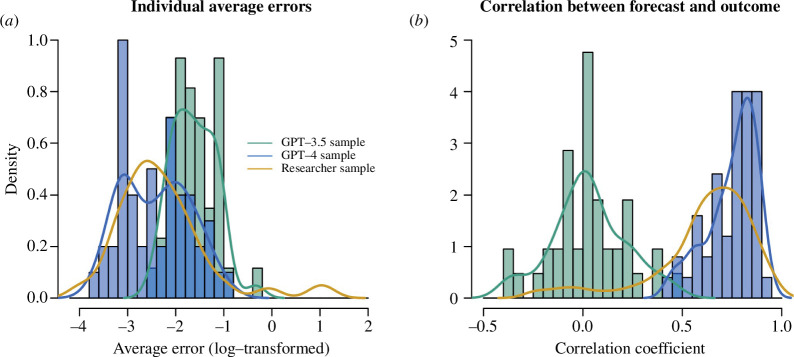
Performance of responses of GPT-3.5 and GPT-4. The LLMs responded to a prompt asking for predictions of the effect sizes in the anger expression study (Tierney W, unpublished data). Forecasts from a cohort of researchers (see (Tierney W, unpublished data)) are shown for comparison. (*a*) Distributions of individual average squared errors. Forecasts from the researcher cohort and GPT-4 display a similar forecasting error, with GPT-4 avoiding very large forecasting errors sometimes made by human forecasters. Errors for the responses by GPT-3.5 tend to be larger. (*b*) Distribution of correlations between individual forecast and observed effect size. Correlations between forecasted and observed effects are typically positive for the researcher cohort, and always positive for GPT-4. For GPT-3.5, the correlations centre around 0.

Performance metrics for GPT-3.5 and GPT-4 as stand-alone forecasting tools (study 1) are shown in figure 1 and table 1. Aggregated effect size forecasts are shown in the electronic supplementary material, table ST1. For GPT-3.5, we observed that responses to the prompts were not always in line with the instructions (see the electronic supplementary material, §6). For several calls the model returned, for instance, 25 rather than 24 values. Of the 50 sampled responses, we excluded seven from further analysis, which either did not consist of 24 values or were not between the bounds of effect sizes of Cohen’s *d* = ± 3 as used for the human cohorts. We retained responses where the format of the output deviated from the instructions; in several responses, for instance, the individual effects were returned as a numbered list despite instructions to omit numbering. All responses, including those excluded from our analyses, are shown in the electronic supplementary material, §6 and §7.

**Table 1.** Average squared errors and correlations with observed outcomes for (*a*) individual forecasts, and (*b*) aggregated forecasts.

(*a*) individual forecasts	average squared errors				correlations with observed effects			
	min	max	median	mean	min	max	median	mean
researcher cohort (*n* = 119)	0.02	3.33	0.09	0.22	−0.24	0.95	0.67	0.61
GPT-3.5 (43 valid runs)	0.08	0.71	0.19	0.22	−0.36	0.45	0.02	0.03
GPT-4 (50 runs)	0.03	0.39	0.09	0.11	0.45	0.91	0.79	0.75
hybrid setting— GPT, initial (*n* = 107)	0.05	4.11	0.27	0.42	−0.29	0.92	0.49	0.43
hybrid setting— GPT, revised (*n* = 107)	0.03	4.88	0.23	0.35	−0.3	0.95	0.59	0.48
hybrid setting— Internet, initial (*n* = 116)	0.06	5.79	0.3	0.57	−0.48	0.91	0.31	0.34
hybrid setting— Internet, revised (*n* = 116)	0.08	4.92	0.28	0.48	−0.58	0.92	0.41	0.37


(*b*) aggregated forecasts	average squared error				correlation with observed effects			
	estimate		95% CI		estimate		95% CI	
researcher cohort	0.02		0.01 .. 0.04		0.87		0.71 .. 0.94	
GPT-3.5	0.1		0.06 .. 0.15		0.07		−0.35 .. 0.46	
GPT-4	0.03		0.01 .. 0.04		0.89		0.76 .. 0.95	
hybrid setting— GPT, initial	0.13		0.09 .. 0.16		0.92		0.82 .. 0.96	
hybrid setting— GPT, revised	0.08		0.06 .. 0.11		0.92		0.82 .. 0.96	
hybrid setting— Internet, initial	0.17		0.12 .. 0.22		0.84		0.65 .. 0.93	
hybrid setting— Internet, revised	0.13		0.09 .. 0.17		0.84		0.67 .. 0.93	

For the 43 included responses from GPT-3.5, there was variation in the returned values, with the standard deviations for predictions for an effect falling in the range between 0.26 and 0.45 (see the electronic supplementary material, table ST2). The average squared errors of the individual forecasts had a median of 0.19 (see table 1 and figure 1*a*); the correlations between returned values and observed effect sizes fell in the range between -0.36 and 0.45, and clustered around 0 (see table 1 and figure 1*b*). Thus, for both metrics, the accuracy was lower compared to the forecasts made by researchers with domain expertise (average squared errors: Wilcoxon rank-sum test, *p* < 0.001; correlation coefficients: two-sample *t*‐test, *p* < 0.001; see also table 1 and the electronic supplementary material, tables ST4 and ST5). Furthermore, an aggregated response that used the average predictions from GPT-3.5 across the 43 valid responses had an average squared error of 0.1, compared to 0.02 for the aggregated response of forecasts from the researchers cohort; and the correlation between the observed effects and aggregated response was 0.07, compared to 0.87 observed for the aggregated responses from the researcher cohort (see table 1 for further details, including 95% confidence intervals (CIs)).

GPT-4 answered all prompts in line with instructions; no responses were excluded from further analysis. As observed for GPT-3.5, there was variation in the returned values, with standard deviations for the individual effect size predictions ranging from 0.13 to 0.64. A robustness analysis (shown in the electronic supplementary material, table ST6), shows that, when using a temperature parameter of 0 rather than the default value of 1, the responses are still stochastic but display less variation. The average squared errors of the individual forecasts (see table 1 and figure 1*a*) obtained under default temperature had a median of 0.09, which is smaller compared to the output from GPT-3.5 (Wilcoxon rank-sum test, *p* < 0.001; for details see §4 and the electronic supplementary material, table ST4), and not statistically significantly different from the forecasts from the researcher cohort (Wilcoxon rank-sum test, *p* = 0.822; for details see §4 and the electronic supplementary material, table ST4). In stark contrast to GPT-3.5, correlations between returned values and observed effects were always positive for GPT-4 and centred around 0.75–0.85 (see table 1 and figure 1*b*). They were statistically significantly larger when compared to GPT-3.5 (two-sample *t*‐test, *p* < 0.001; for details see §4 and the electronic supplementary material, table ST5).

Thus, in summary, for both metrics (correlation with observed effects, and average squared error) GPT-4 responses were more accurate compared to GPT-3.5 and similar in accuracy to the predictions returned by the researcher cohort. Moreover, an aggregated response that used the average predictions from GPT-4 across the 50 repetitions had an average squared error of 0.03, and the correlation between the observed effects and an aggregated response that used the average predictions from GPT-4 prediction was 0.89 (see table 1). Thus, in contrast to GPT-3.5, GPT-4 as a stand-alone tool again displays performance in the forecasting task that is comparable to our cohort of researchers with domain expertise, a finding that is robust with respect to changes in the temperature parameter (see the electronic supplementary material, table ST7). Note that because target gender effects and participant gender effects in the anger expression study were unexpectedly small (Tierney W, unpublished data), the high correlations between forecasted and observed effects observed for GPT-4 and the researcher cohort are largely driven by the variation between the six evaluated characteristics.

### 2.2. Human/AI collaborative forecasting (study 2)

The human/AI collaborative forecasting experiments were conducted at the University of Auckland from 26 Sep to 18 Oct 2023. In total, we recruited 226 participants. Survey data from three participants were excluded, following the preregistered exclusion criteria: in two cases participants accidentally submitted incomplete surveys, and in one case a participant in the Internet setting used AI chatbots. The three excluded participants were in the Internet setting. Of the remaining 223 participants, 116 were in the ChatGPT setting, and 107 in the Internet setting.

**Figure 2.**
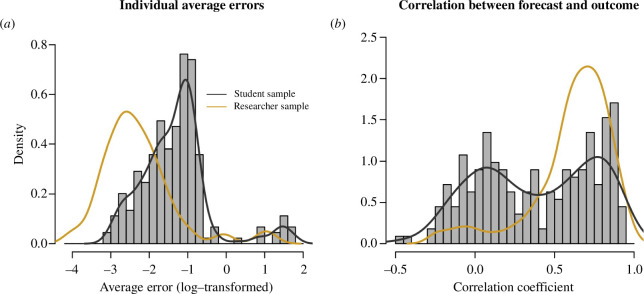
Accuracy of the initial responses of participants from a university subject pool. (*a*) Distributions of individual average squared errors. (*b*) Distribution of correlations between individual forecast and observed effect size. For both metrics, the forecasts from the student cohort tend to be less accurate compared to the forecasts from the researcher cohort. The distribution of correlation coefficients for the student cohort appears to show two peaks, one near 0, and one near the peak observed for the researcher cohort.

Figure 2 shows the distribution of the individual accuracies of the initial forecasts. Compared to the researcher cohort forecasts described earlier, the initial forecasts from the university cohort were less accurate. Average squared errors were larger (median 0.28 vs 0.09; Wilcoxon rank-sum test, *p* < 0.001; for details see §4 and the electronic supplementary material, table ST4) and correlations between forecasted and observed effects lower (mean 0.38 vs 0.61; two-sample *t*‐test, *p* < 0.001; for details see §4 and the electronic supplementary material, table ST5). The distribution of correlations shows a peak near 0, and an additional peak in a range that is close to where correlations from the researcher cohort peaked (coefficients between 0.5 and 1; figure 2*b*). The average squared error of the aggregated initial responses is 0.13 in the GPT setting and 0.17 in the Internet setting (see table 1). However, despite the relatively low accuracy of the individual initial forecasts, the aggregated responses are highly correlated with the observed outcomes (0.92 for the initial responses in the GPT setting and 0.84 for the initial responses in the Internet setting).

**Figure 3.**
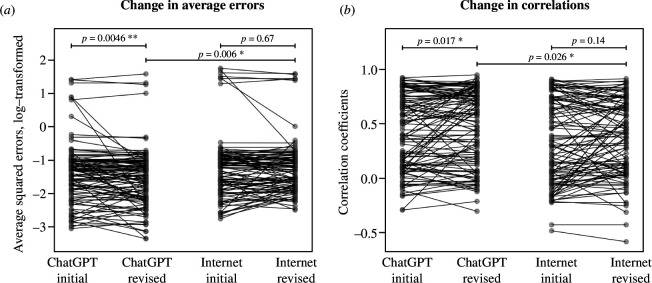
Change of accuracies from initial to revised forecasts. In the ChatGPT setting, revised forecasts are more accurate than initial forecasts, both in terms of smaller average squared errors (*a*) and higher correlations between forecasted and observed effects (*b*) Changes in accuracy in the Internet setting are not statistically different from 0. Revised forecasts from the GPT setting are more accurate compared to revised forecasts from the Internet setting. * denotes *p* < 0.05, ** denotes *p* < 0.005.

The accuracy of the revised responses in the hybrid forecasting is summarized in table 1; the results for the six pre-registered tests (see §4) to compare initial and revised forecasts in the ChatGPT and the Internet settings are visualized in figure 3. In the ChatGPT setting, the participants’ average squared forecasting error decreased after revisions (median 0.27 for the initial vs 0.23 for the revised forecasts). The decrease is statistically significant (Wilcoxon signed rank test, *V* = 3670, *p* = 0.0046). In the Internet setting, the participants’ average squared forecasting error also decreased after revisions (median 0.30 for the initial vs 0.28 for the revised forecasts) but the decrease is not statistically significant (*V* = 2596, *p* = 0.67). Furthermore, in the ChatGPT setting, the participants’ correlations between forecasted and observed effects increased after revisions (mean 0.43 for initial vs 0.48 for revised forecasts), with suggestive evidence for the increase (*t*_115_ = −2.43, *p* = 0.017). In the Internet setting, the participants’ correlations between forecasted and observed effects increased after revisions (mean 0.34 for initial vs 0.37 for revised forecasts) but again the increase is not statistically significant (*t*_106_ = −1.50, *p* = 0.14).

Comparing across the settings, we find suggestive evidence that revised forecasts from the ChatGPT setting had smaller average squared errors than the revised forecasts from the Internet setting (*W* = 4874, *p* = 0.006). Similarly, we find suggestive evidence that the revised forecasts from the ChatGPT setting had higher correlations with observed effects, compared to the revised forecasts from the Internet setting (*t*_219.27_= 2.24, *p* = 0.026).

While the above analyses provide suggestive evidence for more accurate revised forecasts in the GPT setting compared to the Internet setting, and a statistically significant improvement of the accuracies for the ChatGPT setting but not the Internet setting, the improvements in accuracy do not differ significantly between the settings (rank sum tests on reduction of error: *W* = 5432, *p* = 0.11; *t*-tests for increases in correlation: *t*_207.37_= 0.35, *p* = 0.72; note that these tests were not pre-registered). Thus, while we observe evidence for improved forecasts in the ChatGPT setting, there is no conclusive evidence that the improvements in the ChatGPT setting exceed the improvements in the Internet setting.

We find no statistically significant differences in the average squared forecasting errors for the initial forecasts between male and female forecasters, and no effects for political orientation, or gender system justification (see the electronic supplementary material, table ST3). However, there is suggestive evidence of effects for forecaster gender (estimate: 0.12, *p* = 0.017 for female vs male forecasters) and political orientation (estimate: − 0.048, *p* = 0.005 with increasing conservative views on a seven-point scale) on the correlation between initial forecasts and observed outcomes. Overall, male, and politically more liberal forecasters exhibited higher correlations between predictions and the observed results. However, given the lack of any evidence of an effect of these variables on the average squared forecasting error, these results should be interpreted cautiously.

## 3. Discussion

Our results demonstrate that there is potential for LLMs as stand-alone tools to generate accurate predictions for the effects from studies in the social and behavioural sciences. Remarkably, GPT-4 performed approximately as well as a cohort of human experts in predicting the overall results of a highly complex crowdsourced many-designs and many-labs research project. The next step towards establishing the general use of AI in forecasting contexts is to examine how well our findings generalize to other sorts of research questions and research designs. Ideally, such a test should rely on predictions that are made before an experiment is conducted. Such an approach ensures that not only the model is ‘blind’ to the results (i.e. the results are not part of the data used to train the models), but also the researchers who design the prompts. The latter is important because forecasts can be influenced (inadvertently or not) through prompt design. Although in our study the researchers who designed the prompts knew the results, we aimed to minimize such an influence by committing to a single prompt.

The contrasting results for GPT-3.5 and GPT-4 show that the performance in forecasting empirical effects in the social and behavioural sciences can differ substantially between models, and that it may continue to improve into the future. Our findings are in line with earlier performance evaluations [34–37], but we can only speculate about the technological changes behind the observed improvement: the increased context window size, together with the larger model size, are plausible factors. It remains to be investigated if finetuning of LLMs with data from scientific publications and more complex strategies in prompt design, such as chaining-of-thought prompting and self-consistency leveraging strategies [38,39], can further improve forecasting accuracy.

The responses of the LLMs to our prompts show considerable variation which is influenced by the temperature parameter (see the electronic supplementary material, tables ST6 and ST7). Interestingly, the ensemble of GPT-4 responses displays ‘wisdom-of-the-crowds’ in that the aggregated response is about as accurate as its most accurate individual forecast, which has also been observed for forecasting in other domains [19]. It remains to be investigated if the accuracy of aggregated ‘wisdom-of-crowds’ LLM responses depends on the temperature parameter.

Our results from study 2 indicate that laypeople can improve the quality of their individual forecasts of scientific results by collaborating with AI. There was a substantial difference between the accuracies of forecasts from researchers with domain expertise, and forecasts from the cohort of participants at Auckland University, which consists of undergraduate and graduate students. There is also considerable variation in the accuracy of the forecasts within the student cohort probably stemming from varied skills in predicting human behaviour, and different abilities in effectively using AI tools [25], and this probably translates into a variety of mechanisms that could be at play to improve forecasts in the ChatGPT setting. It remains to be analysed what these mechanisms are, and it also remains to be studied if individual researchers with domain expertise can improve their scientific predictions with AI tools. The human/AI interaction we offered in the ChatGPT setting allowed for unstructured dialogue, and thus participants could adjust their interaction with the AI to their preferences and requirements. To identify mechanisms that contributed to an increased accuracy, a more standardized interaction between human participants and AI, and a larger sample of participants will be of advantage.

We believe that both stand-alone AI tools and hybrid human–AI collaborations will prove impactful in this space in the coming years. Indeed, we see potential for AI to enhance multiple stages of the scientific process. Several commercial services have already emerged to use AI tools for literature searches and information retrieval from individual publications. At the analysis stage, it has been argued that machine intelligence can potentially help address the ongoing crisis of confidence in science [40] by identifying replicable, robust, and generalizable empirical patterns absent of bias based on intellectual or ideological pre-commitments [41]. On the other hand, there are reasons to be cautious when using AI tools for decision-making in science. Given LLMs are trained based on human communication, there is reason for concerns that AIs reinforce and amplify human biases [42]. Moreover, since LLMs are not always accurate, and the domains for which they can provide accurate forecasts are not well established, over-reliance [43,44] on LLMs to predict effects could well be detrimental to science. For now, AIs remain more proficient at detecting patterns than at explaining them in theoretical terms. However, the rapid advances in AI—note the dramatic improvement in forecasting precision between GPT-3.5 and GPT-4 in the present study 1—raises the possibility that a cautious use of AI tools may soon contribute meaningfully at the idea generation and theoretical explanation stages as well.

The present findings highlight ways in which AI tools may add value to early stage scientific workflows currently carried out by humans. For example, using AI to anticipate whether a scientific hypothesis will find empirical support could help allocate limited research resources more efficiently. In our study, the LLM was no more accurate than the crowd of human scholars. However, a chatbot can be queried endlessly with minimal effort, making it far easier to scale up to complement nearly any replication or original study design. By contrast, crowds of human experts cannot realistically be recruited repeatedly, and they will only be able and willing to submit a comparatively limited number of forecasts at any given point in time. With LLMs and other AI tools becoming more accurate, and their capabilities and limitations better understood, they are likely to play an increasing role in empirical research.

## 4. Methods

### 4.1. Study 1: forecasts generated by GPT-3.5 and GPT-4

To query GPT-3.5 and GPT-4 for forecasts about the effects in the anger expression study, we used a prompt derived from the survey used for the researcher cohort in the forecasting part of the anger expression study (see Supplement 17 in (Tierney W, unpublished data) and the electronic supplementary material). For the prompt, we focused on 24 questions from the survey, removed repetitions in the instructions and avoided technical language. We omitted 12 questions from the original survey because they did not elicit predictions for between-condition effects. We started by submitting all 24 questions on 4 July 2023, to ChatGPT (model GPT-3.5) in a single prompt, without any further adjustments, using the OpenAI chatbot user interface from a personal account of the corresponding author. The chatbot returned 24 values along with some commentary (see the electronic supplementary material, S2 for prompt and response). The same query was sent on 12 September 2023, to GPT-4 using the OpenAI application programming interface (API), and chatbotui.com as the user interface (see the electronic supplementary material, S3 for the response). To further investigate the responses of GPT-3.5 and GPT-4, we then submitted the prompt 50 times to each of the two models gpt-3.5-turbo-0613 and gpt-4-0613, using the R library ‘httr’ [45] and default model parameters. Additionally, we ran gpt-4-0613 50 times with temperature parameters of 0, 0.2, 0.6 and 1.0 (default), respectively, for a robustness analysis shown in the electronic supplementary material. The original prompt was modified with the objective to receive the 24 numerical effect size estimates in a standardized format without additional text commentary (see the electronic supplementary material, S5–S7 for the prompt and all responses). We used GPT-4 model gpt-4-0613 even though updated models (including GPT-4-turbo) became available in November 2023. The models gpt-3.5-turbo-0613 and gpt-4-0613 have the same training data cut-off date (September 2021). The context window is 4 096 tokens for gpt-3.5-turbo-0613, and 8 192 tokens for gpt-4-0613.

We excluded responses from further analysis, that either did not consist of 24 values or were not between the bounds of effect sizes of Cohen’s *d* = ± 3 as used for the human cohorts. We retained responses where the format of the output deviated from the instructions. While the timing of the study makes it unlikely that results from the anger expression study could have contaminated [46] the training data of the models, we used the following three approaches to find indications for such a contamination: first, we used five of the attributes from the anger expression study (competence, warmth, being ‘out-of-control’, dominance, assertiveness and status), and asked the LLM to complete the sixth (using, for instance the prompt: ‘add one more attributes to the following list: assertiveness, out-of-control, dominance, competence, warmth’); we observed that the model never responds with the missing attribute. Second, when asked for three recent publications investigating gender effects in the perception of individuals who express anger, the model never listed the study used for the forecasting task. Finally, in study 2, the LLM identified earlier publications on the topic, but it never pointed to the actual target study. Note that the use of the API in studies 1 and 2 prevents indirect leakage via earlier prompts. All responses, including those excluded from our analyses, are shown in the electronic supplementary material, §6 and §7.

### 4.2. Study 2: human/AI collaborative forecasting

In our human/AI collaborative forecasting experiment, we investigated whether a GPT-4 powered chatbot can help forecasters from a university subject pool to predict the outcomes of the 24 effects from the anger backlash study. We investigated two different experimental conditions, labelled ‘ChatGPT setting’ and ‘Internet setting’. Both settings consisted of two stages. In the ChatGPT setting, participants received initial instructions and then were asked to provide an initial set of 24 predictions that are made without any aid beyond the information provided in the instructions. Participants had 25 min to read the instructions and complete their initial predictions. In the second stage, participants could use a GPT-4 powered chatbot to revise their predictions. They had an additional 25 min to complete this task, and faced no constraints in how they queried the language model. We used chatbotui.com as a user interface together with an OpenAI API key to provide access to GPT-4 (see the electronic supplementary material). The Internet setting was structured in the same way, but in stage 2, participants could use internet resources such as web searches. Participants in the Internet setting were not allowed to use ChatGPT or other AI chatbots. Their Internet browser was set to www.google.com. The survey to enter the initial and revised predictions was implemented in Qualtrics and concluded with a brief demographic section with questions covering gender, political orientation (conservative-liberal), and gender system justification [47]. Instructions and Qualtrics surveys are available on the Open Science Framework (OSF; osf.io/g5zcx).

Participants were recruited through ORSEE [48] from a subject pool that is open to the public and consists largely of undergraduate and postgraduate students. Participation was in person at the DECIDE laboratory at the University of Auckland. Participants received a fixed payment of NZD 20 for participating in the experiment; one in four participants was randomly selected to receive a bonus of up to NZD 25 that was calculated as NZD max(0.25-(*SqErr* * 25)), with *SqErr* being the average over the squared error of one randomly selected effect from the initial survey and one randomly selected effect from the revised survey. We set the target sample size to a minimum of 110 participants in each setting, and we stopped recruiting once this number had been reached. Our target was informed by constraints in recruitment from a finite subject pool within a limited time span towards the end of the New Zealand academic year.

### 4.3. Pre-registered analyses, access to data and scripts and related manuscripts and datasets

The analysis of the data from the human/AI collaborative forecasting study was pre-registered on OSF (osf.io/g5zcx) and was conducted as described in the pre-registration. We pre-registered to use two metrics to evaluate the accuracy of the forecasts of an individual: the average squared error over the 24 forecasts an individual made; and the Pearson correlation coefficient between the 24 forecasted and observed effect sizes. The average squared error between forecasted and observed effects is well-suited to quantify accuracy for these continuous variables and has also been used to evaluate the predictions from the researcher cohort (Tierney W, unpublished data). The Pearson correlation between forecasted and observed effects is used as a second accuracy metric because it is robust with respect to participants’ potential difficulties in judging the overall magnitude of experimental effects. For all our analyses, we use two-sided *p*-values.

We pre-registered to use both the more conservative significance threshold of *p* < 0.005 proposed by Benjamin *et al*. [49], and the traditional threshold for statistical significance of *p* < 0.05. We refer to evidence that meets the traditional threshold of *p* < 0.05 but not the threshold of *p* < 0.005 as suggestive evidence. Given the diversity of opinions regarding the use of thresholds for statistical inference, we invite readers to follow their own preferences when interpreting our results.

Our design allows us to conduct within-subject comparisons of the accuracies of the participants’ initial and revised responses and between-subject comparisons of the responses in the two different treatment groups. To analyse whether revised forecasts differ in their accuracy from the initial forecasts, we pre-registered to use a Wilcoxon signed-rank test between the participants’ average squared error of the initial forecast and the participants’ average squared error of the revised forecast. The test was separately conducted for the ChatGPT condition and the Internet condition; a Wilcoxon signed-rank test was pre-registered because we expected a considerable deviation of the average error from a normal distribution. Moreover, we pre-registered paired *t*-tests comparing the participants’ correlation coefficients between initial forecast and observed effect sizes and the correlation coefficients between revised forecast and observed effect sizes. As for the tests on forecasting errors, this test was conducted separately for the ChatGPT setting and the Internet setting.

In addition, we pre-registered two tests to analyse whether the revised forecasts from the two settings differed: a Wilcoxon rank-sum test to compare average squared errors for the participants from the ChatGPT setting with the average squared errors for participants from the Internet setting, and a two-sample independent *t*‐test between the correlation coefficients for the participants from the ChatGPT setting and the participants from the Internet setting. Finally, we pre-registered two analyses to test whether the accuracies of the initial predictions vary by forecaster gender, political orientation, and views on gender justice.

We use an additional analysis to compare improvements in accuracy between the ChatGPT setting and the Internet setting in study 2. This analysis was not pre-registered.

We did not pre-register any analyses of GPT-3.5 and GPT-4 as stand-alone tools (study 1) because the research ideas behind this part of the project developed during the data analysis of the human/AI collaborative forecasting experiments. (Because the ease at which these data can be generated, a pre-analysis plan ahead of data collection does not increase the credibility of our results in this case, as it is not verifiable that predictions were not sampled prior to the pre-registration.) To test for the differences in accuracy between the responses of GPT-3.5, GPT-4, the researcher cohort and the student cohort, we follow analytic approaches equivalent to those used for the human/AI collaborative forecasting study. Specifically, we used pairwise Wilcoxon rank-sum tests to compare average squared errors and pairwise two-sample independent *t*‐tests for the correlation coefficients. The results of these tests that were not pre-registered are shown in the electronic supplementary material, tables ST4 and ST5.

Throughout the study, we committed to using the same single prompt, with minimal modifications as outlined in the Methods throughout study 1. All data from studies 1 and 2, and scripts used for analysis and visualization are available on OSF [50]. Results of the anger expression study, including the 24 effects that are focus of this study and the forecasts from 119 researchers with domain-expertise are available in (Tierney W, unpublished data), with the data available at [51].

## Data Availability

All data from studies 1 and 2, and scripts used for analysis and visualization are available on OSF [50]. Results of the anger expression study, including the 24 effects that are focus of this study and the forecasts from 119 researchers with domain-expertise are included in this submission as additional information, with the data available at [51]. Supplementary material is available online [52].
